# Effect of the Spencer Technique on Glenohumeral Joint Range of Motion: A Comparative Analysis of Athletes Versus Non-athletes

**DOI:** 10.7759/cureus.91863

**Published:** 2025-09-08

**Authors:** Daniel P Oar, Alexandria Zarilla, Dante DiSilvestro, Zachary J Buchman, Alex Abouafech, David Boesler

**Affiliations:** 1 School of Medicine, Lake Erie College of Osteopathic Medicine, Bradenton, USA; 2 Osteopathic Medicine, Lake Erie College of Osteopathic Medicine, Bradenton, USA

**Keywords:** athletic performance, glenohumeral joint, omt in sport, orthopedic sports medicine, osteopathic manipulative medicine, osteopathic manipulative treatment (omt), spencer technique

## Abstract

Introduction: The Spencer technique of the shoulder is an osteopathic manipulative technique used to treat somatic dysfunction and improve glenohumeral range of motion. Current literature aims to examine the efficacy of the Spencer technique of the shoulder, but falls short in investigating the technique's usefulness in patient populations with varying levels of physical activity and joint usage. This study examines the Spencer technique’s impact on the shoulder range of motion in recreational pickleball players compared to non-pickleball players.

Objective: The goal of this study was to compare the effects of the Spencer technique on the shoulder in recreational pickleball players versus non-pickleball players.

Methods: Fifty-four participants were recruited and divided into four groups: pickleball players with Spencer treatment (n=12), pickleball players with no Spencer treatment (n=12), non-pickleball players with Spencer treatment (n=15), and non-pickleball players with no Spencer treatment (n=15). The Spencer treatment groups were treated twice per week for two weeks, and the pickleball playing groups participated in recreational pickleball sessions twice per week for two weeks. Measurements of shoulder range of motion in flexion, extension, internal rotation, external rotation, abduction, and adduction were taken at study onset and two weeks later upon study completion. The average change in range of motion from baseline for all four groups was determined, and two-factor analyses of variance (ANOVAs) with post-hoc two-sample, two-tailed t-tests were used to investigate individual and combined effects as well as differences amongst the groups in each plane of motion.

Results: Of the initial 54 participants enrolled in the study, 53 successfully completed all study requirements and were included in the data analysis. Statistically significant differences were found amongst the four different groups in terms of average change in degrees of shoulder flexion, extension, abduction, internal rotation, and external rotation over the two-week course of the study. This difference was seen when comparing the pickleball and Spencer treatment group, which experienced greater change, to the other groups in flexion, extension, abduction, internal rotation, and external rotation. No significant differences were observed amongst the four groups for shoulder adduction.

Conclusion: This study found that Spencer treatment paired with pickleball playing produced a statistically significant greater change in range of motion from baseline for shoulder flexion, extension, abduction, internal rotation, and external rotation compared to the other groups. No significant differences in shoulder adduction were found amongst the four groups. These findings highlight the benefit of combining Spencer treatment with recreational exercise in improving shoulder range of motion. With a more complete understanding of how Spencer treatment affects patients with varying exercise habits, osteopathic clinicians will be better positioned to deliver more personalized care and ensure optimal clinical outcomes.

## Introduction

Osteopathic manipulative treatment (OMT) has long been known to be efficacious in treating and preventing injuries, improving musculoskeletal function, and improving mobility in a wide variety of populations, from sedentary individuals to elite athletes [1-3]. The Spencer technique is an OMT technique that can be used on the glenohumeral joint or the femoroacetabular joint in order to improve joint mobility or address myofascial restrictions [4]. The Spencer technique of the shoulder is performed by bringing the shoulder joint to its restrictive barrier while applying an articulatory technique, using repetitive, rhythmic movements into the restrictive barrier [4]. This technique is done through eight sequential steps: flexion, extension, circumduction with compression, circumduction with traction, abduction, adduction, internal rotation, and distraction/pumping fluid [4]. Although there have been studies investigating the use of the Spencer technique of the shoulder in athletes, there is little research exploring the use of the Spencer technique on recreational athletes using their shoulder joint for competition, such as pickleball players, and comparing the results directly to a group of sedentary individuals [2]. However, there have been studies conducted on the use of the Spencer technique of the hip on runners with varying results [5,6]. This necessitates a comprehensive study on the use of the Spencer technique of the shoulder in recreational athletes versus non-athletes to fill the gap in the existing literature.

Through repetitive overhand and underhand multidirectional movements requiring significant upper extremity coordination, pickleball served as the form of recreational exercise involving glenohumeral joint movement in this study. Other racquet sports, such as tennis, have been proven to require dynamic activation of the rotator cuff, scapular stabilizers, and glenohumeral musculature, promoting neuromuscular adaptations [7]. Pickleball can therefore be hypothesized to alter musculoskeletal restrictions and physical capacity [7,8]. Additionally, pickleball has quickly become one of the fastest-growing sports in the United States [9]. According to the Association of Pickleball Players 2023 participation report, nearly 48.3 million people had played pickleball in the last year [9]. Due to its recent rise in popularity and the significant glenohumeral joint usage required to play, it is a good representation of a common recreational sport with significant glenohumeral joint implications, validating its use as an intervention in this study.

Shoulder pain is a common musculoskeletal complaint, with a lifetime prevalence of up to 66.7% in the general population [10]. In addition, while shoulder pain is often self-limiting, up to half of those experiencing shoulder pain continue to have pain or functional disturbances up to two years after initial symptoms [10]. These statistics indicate a need for alternative methods of improving and maintaining shoulder range of motion (ROM) and stability while decreasing pain. A study comparing the Spencer technique of the shoulder versus passive stretching in those with adhesive capsulitis found that the Spencer technique was superior at reducing pain, improving joint ROM, and improving functionality of the joint [11]. A similar study by Amin et al. concluded that the Spencer technique was more effective than standard physical therapy at reducing pain and improving joint function in patients with adhesive capsulitis [12]. Knebl et al. evaluated the use of the Spencer technique on an elderly population with preexisting shoulder problems [13]. It was found that only those treated with the Spencer technique showed continued improvement following treatment, while those in the control group regressed over time [13]. These findings demonstrate the long-term benefits of the Spencer technique and highlight an avenue for continued symptom relief and improvement in functionality. A study involving collegiate baseball players found that the use of the Spencer technique of the shoulder counteracted the negative effects of repeated overhand throwing on internal rotation and abduction [2]. This study demonstrated the impacts of the Spencer technique specifically on collegiate athletes, serving as inspiration for this study to compare recreational pickleball players to non-players.

With current literature regarding the efficacy of the Spencer technique in mind, we aimed to compare changes in ROM between recreational athletes and non-athletes receiving the Spencer technique. This study aims to fill the gap in the existing literature and directly compare the effectiveness of the Spencer technique on these two groups to gain a better understanding of the clinical utility of the Spencer technique. We hypothesize that those who engage in recreational exercise that activates the glenohumeral joint, such as pickleball, in addition to receiving the Spencer technique, will show greater improvements in ROM compared to the other groups in the study due to musculoskeletal adaptations of the glenohumeral joint that may occur from increased usage during recreational athletics.

## Materials and methods

To evaluate the impact of the Spencer technique on the shoulder on glenohumeral ROM, an Institutional Review Board (IRB) approval was requested and granted to carry out this study. Upon IRB approval, this study was assigned the number 32-107. This study was funded using the researchers’ personal funds. Informed consent was obtained from all participants prior to the start of the study once initial screening requirements were met. Informed consent documents were completed on a hard copy paper form and then stored for future reference. This informed consent document explained the study in full, including the purpose of the study, inclusion and exclusion criteria, potential risks and benefits, confidentiality, voluntary participation, voluntary and involuntary withdrawal, and procedures over the course of the study. Additionally, all prospective participants were given the opportunity to ask questions prior to signing the document and enrolling in the study. Signatures of all participants and investigators were then obtained. Participants were compensated through a gift card raffle where two participants were selected at random to receive a $50.00 Amazon.com gift card.

Inclusion and exclusion criteria

Inclusion criteria required participants to be current first- or second-year medical students at Lake Erie College of Osteopathic Medicine (LECOM), Bradenton, attend both ROM measurement sessions, attend 100% of treatment sessions if applicable, and attend 100% of pickleball playing sessions if applicable. Exclusion criteria included being unable to sign an informed consent, having a past medical history of a shoulder injury of any kind, or having a serious health condition of any kind that prevents participation in vigorous exercise. In order to be successfully enrolled, potential participants had to agree to complete, or be in compliance with, all the inclusion criteria and none of the exclusion criteria.

First- and second-year medical students were recruited to participate in this study via email. Interested students responded to the email via an attached Google Form, and potential participants were then screened according to the inclusion and exclusion criteria. Those eligible to participate in the study were selected and invited to attend an initial ROM assessment. A total of 54 participants were invited to attend the initial ROM assessment and agreed to sign consent forms, officially enrolling them in the study.

After all participants were officially enrolled in the study, initial baseline shoulder ROM measurements were taken. Shoulder flexion, extension, abduction, adduction, internal rotation, and external rotation of the participants’ dominant arm were recorded in degrees using a hand-held goniometer. After initial shoulder ROM measurements were taken, 24 participants were assigned to play recreational pickleball twice weekly for two weeks, for a total of four individual playing sessions (n=24). The remaining 30 participants were assigned to abstain from playing pickleball for the following two weeks (n=30). Group assignments were determined based on each participant's willingness to participate in pickleball playing sessions. Participants were additionally randomly assigned to receive treatment (n=27) or to receive no treatment (n=27). This resulted in four distinct groups consisting of pickleball playing and treatment (P+T) (n=12), pickleball playing and no treatment (P+NT) (n=12), no pickleball playing and treatment (NP+T) (n=15), and no pickleball playing and no treatment (NP+NT) (n=15). Participants selected to the treatment groups received the Spencer technique of the shoulder, on their dominant arm, twice weekly for two weeks, for a total of four individual treatments. With the exception of restricted pickleball playing for those in the non-pickleball groups, all participants were instructed to maintain their normal activity level throughout the course of the study.

Treatment of all study subjects was provided by the research team under the supervision of an experienced osteopathic physician. To ensure all members of the research team were proficient in providing quality and consistent treatments, all investigators demonstrated the technique, in accordance with Nicholas’ Atlas of Osteopathic Techniques, to the supervising osteopathic physician until deemed competent to treat study subjects [4]. To ensure equal and effective treatment was provided, the Spencer technique was performed in a standardized fashion using the sequential steps outlined in Table 1.

**Table 1:** Protocol of the Spencer technique of the shoulder used in this study Each step was performed on the patients’ dominant arm and as adherently as possible to the directions provided in Nicholas’ Atlas of Osteopathic Techniques [4]. For all stages, the patient was in the lateral decubitus position with their dominant shoulder facing up. Each step was done once at each treatment session. Standardized procedures were required to be implemented as there is variation in terms of the acceptable number of thrusts and circumductions per stage. This table was created independently by the authors using Nicholas’ Atlas of Osteopathic Techniques as a reference [4].

Stages	Steps
Stage 1: Extension	1. The physician stands next to the shoulder being treated.
	2. The physician flexes the elbow and carries the shoulder to the extension restrictive barrier, where five articulatory springing motions are applied at the end range of motion while stabilizing the joint with the physician’s other hand.
Stage 2: Flexion	1. The physician stands next to the shoulder being treated.
	2. The physician extends the elbow and carries the shoulder to the flexion restrictive barrier, where five articulatory springing motions are applied at the end range of motion while stabilizing the joint with the physician’s other hand.
Stage 3: Circumduction With Compression	1. The physician stands next to the shoulder being treated.
	2. The physician flexes the elbow and applies a compressive force down through the glenohumeral joint while stabilizing the joint with the physician’s other hand.
	3. The physician circumducts the patient’s shoulder through five small and enlarging circles (clockwise, then counterclockwise directions) while maintaining compression.
Stage 4: Circumduction With Traction	1. The physician stands next to the shoulder being treated.
	2. The physician extends the elbow and applies a traction force up through the glenohumeral joint while stabilizing the joint with the physician’s other hand.
	3. The physician circumducts the patient’s shoulder through five small and enlarging circles (clockwise, then counterclockwise directions) while maintaining traction.
Stage 5: Abduction	1. The physician stands next to the shoulder being treated.
	2. The physician carries the shoulder to the abduction restrictive barrier, where five articulatory springing motions are applied at the end range of motion while stabilizing the joint with the physician’s other hand.
Stage 6: Adduction	1. The physician stands next to the shoulder being treated.
	2. The physician carries the shoulder to the adduction restrictive barrier, where five articulatory springing motions are applied at the end range of motion while stabilizing the joint with the physician’s other hand.
Stage 7: Internal Rotation	1. The physician stands next to the shoulder being treated.
	2. The physician places the patient’s hand behind their back and carries the shoulder to the internal rotation restrictive barrier, where five articulatory springing motions are applied at the end range of motion while stabilizing the joint with the physician’s other hand.
Stage 8: Distraction/Pumping Fluid	1. The physician stands next to the shoulder being treated.
	2. Gentle compression and release movements are applied to the glenohumeral joint subcutaneous tissues to encourage lymphatic drainage.

**Figure 1:**
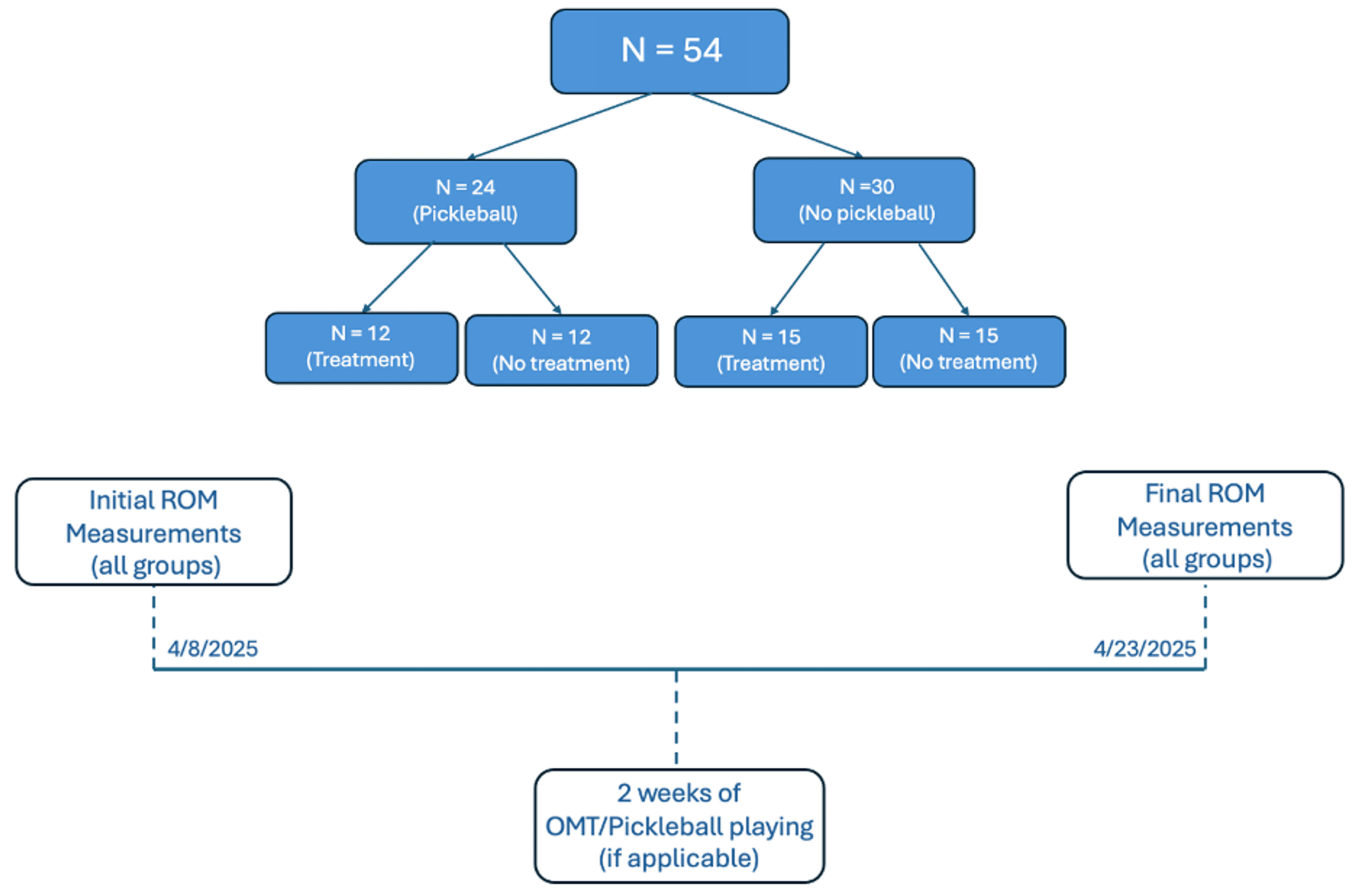
Group stratification and study timeline with associated dates of each event ROM: range of motion; OMT: osteopathic manipulative technique

Following the two weeks of treatment and pickleball playing, final ROM measurements on the participants’ dominant arm were taken. These measurements were obtained one day following the last Spencer treatment performed on the treatment groups. A set of pre- and post-treatment and recreational pickleball playing ROM measurements was therefore obtained and available for interpretation and data analysis. A summary of the timeline used for this study is shown in Figure 1.

For both times data was collected, ROM in degrees of flexion, extension, abduction, adduction, internal rotation, and external rotation was measured in the participants’ dominant arm. This resulted in final data on ROM in flexion, extension, abduction, adduction, internal rotation, and external rotation measured at two distinct points in time, both at the start of the study at initial ROM assessments (April 8, 2025, 12:00 pm) and at the conclusion of the study at final ROM assessments (April 23, 2025, 12:00 pm).

This allowed for the average change in degrees for each plane of motion, between both points in time, to be determined. Unbalanced two-factor analyses of variance (ANOVAs) with replication were performed to compare the average degrees of change from initial to final ROM for shoulder flexion, extension, abduction, adduction, internal rotation, and external rotation amongst the four groups and investigate individual and combined effects. Further, post-hoc two-sample, two-tailed T-tests were completed to identify individual differences between the specific groups. All data analysis was performed using Google Sheets (Google, Inc., Mountain View, CA, USA) via the XLMiner Analysis ToolPak extension (Frontline Systems Inc., Incline Village, NV, USA).

## Results

Of the initial 54 total participants enrolled in the study, 53 participants successfully completed all study requirements, including attending both ROM measurement sessions, all pickleball playing sessions, if applicable, and all Spencer technique treatment sessions, if applicable. Only one of the initial 54 participants was lost to follow-up over the course of the study, and this person’s data was not included in our analysis. This individual was a member of the P+NT group. This left a total of 12 participants in the P+T group, 11 participants in the P+NT group, 15 participants in the NP+T group, and 15 participants in the NP+NT group. All participants enrolled in both treatment groups had a 100% treatment attendance rate, and all participants enrolled in both pickleball playing groups had a 100% pickleball participation rate, with the exception of one participant lost to follow-up.

To compare the average change in shoulder flexion ROM over the course of the study, Table 2 displays the results of an unbalanced, two-factor ANOVA with replication, and Table 3 displays post-hoc two-sample, two-tailed t-tests, comparing average change in shoulder flexion between the individual groups. The results of this ANOVA demonstrate that pickleball, as an effect by itself, did increase flexion, regardless of concurrent treatment (p=0.0410), and Spencer treatment, as an effect by itself, did increase flexion, regardless of concurrent treatment (p=0.0460). When examining the interaction of the two treatments, the presence of pickleball did not affect the Spencer treatment in a non-additive manner, and vice versa (p=0.0630), in regards to flexion. Post-hoc t-tests revealed that a difference was present between the P+T group (average change of 24.50 (±16.37) degrees of flexion) versus P+NT group (average change of 6.81 (±14.87) degrees of flexion), a difference of 17.69 degrees, p=0.0133; P+T group (average change of 24.50 (±16.37) degrees of flexion) versus NP+T group (average change of 7.80 (±13.27) degrees of flexion), a difference of 16.70 degrees, p=0.0071; and P+T group (average change of 24.50 (±16.37) degrees of flexion) versus NP+NT group (average change of 6.20 (±16.36) degrees of flexion), a difference of 18.30 degrees, p=0.0079. No significant difference in the average change of degrees of shoulder flexion was found between any of the other groups. These findings demonstrate a significant difference in the average change of degrees of shoulder flexion between the P+T group compared to all three other groups. This suggests that exercise involving the glenohumeral joint with recreational pickleball in combination with the use of Spencer treatment shows significantly greater improvements in degrees of shoulder flexion compared to playing recreational pickleball, or receiving Spencer treatment in isolation, or receiving neither intervention.

**Table 2:** Results of an unbalanced, two-factor ANOVA with replication analyzing each intervention’s impact and interaction on shoulder flexion * signifies a statistically significant difference, (p<0.05); SD: standard deviation; ANOVA: analysis of variance

Group	Average Change of Flexion (Degrees) Over the Course of Study	Variance	df	F	F-crit	Effect P-value
Yes Pickleball + Yes Spencer Treatment	24.5 (±16.37)	267.91	1.00	4.40	4.04	0.0630
Yes Pickleball + No Spencer Treatment	6.81 (±14.87)	221.16	1.00	4.19	4.04	0.0410*
No Pickleball + Yes Spencer Treatment	7.8 (±13.27)	176.03	1.00	3.62	4.04	0.0460*
No Pickleball + No Spencer Treatment	6.2 (±16.36)	267.74	-	-

**Table 3:** Post hoc two-sample, two-tailed t-test comparing the average change in shoulder flexion over the course of the study between the individual groups * signifies a statistically significant difference (p<0.05) Group 1: Yes Pickleball + Yes Spencer Treatment; Group 2: Yes Pickleball + No Spencer Treatment; Group 3: No Pickleball + Yes Spencer Treatment; Group 4: No Pickleball + No Spencer Treatment

Between Which Groups	1 vs. 2	1 vs. 3	1 vs. 4	2 vs. 3	2 vs. 4	3 vs. 4
p-value	0.0133*	0.0071*	0.0079*	0.86	0.92	0.77
t-value	2.82	2.76	2.75	-0.26	-0.23	0.03

To compare the average change in shoulder extension ROM over the course of the study, Table 4 displays the results of an unbalanced, two-factor ANOVA with replication, and Table 5 displays post-hoc two-sample, two-tailed t-tests, comparing average change in shoulder extension between the individual groups. The results of this ANOVA demonstrate that pickleball, as an effect by itself, did increase extension, regardless of concurrent treatment (p=0.0022), and Spencer treatment, as an effect by itself, did not increase extension, regardless of concurrent treatment (p=0.16). When examining the interaction of the two treatments, the presence of pickleball did not affect the Spencer treatment in a non-additive manner, and vice versa (p=0.35), in regards to extension. Post-hoc t-tests revealed that a difference was present between the P+T group (average change of 24.50 (±14.16) degrees of extension) versus P+NT group (average change of 13.63 (±7.38) degrees of extension), a difference of 10.87 degrees, p=0.0335; P+T group (average change of 24.50 (±14.16) degrees of extension) versus NP+T group (average change of 6.27 (±19.72) degrees of extension), a difference of 18.23 degrees, p=0.0125; P+T group (average change of 24.5 (±14.16) degrees of extension) versus NP+NT group (average change of 3.73 (±17.09) degrees of extension), a difference of 20.77 degrees, p=0.0024. No significant difference in the average change of degrees of shoulder extension was found between any of the other groups. These findings demonstrate a significant difference in the average change of degrees of shoulder extension between the P+T group compared to all three other groups. This suggests that exercise involving the glenohumeral joint with recreational pickleball in combination with the use of Spencer treatment shows significantly greater improvements in degrees of shoulder extension compared to playing recreational pickleball, or receiving Spencer treatment in isolation, or receiving neither intervention.

**Table 4:** Results of an unbalanced, two-factor ANOVA with replication analyzing each intervention’s impact and interaction on shoulder extension * signifies a statistically significant difference (p<0.05); SD: standard deviation; ANOVA: analysis of variance

Group	Average Change of Extension (Degrees) Over Course of Study	Variance	df	F	F-crit	Effect P-value
Yes Pickleball + Yes Spencer Treatment	24.5 (±14.16)	200.45	1.0	10.42	4.04	0.35
Yes Pickleball + No Spencer Treatment	13.63 (±7.38)	54.45	1.0	1.99	4.04	0.00220*
No Pickleball + Yes Spencer Treatment	6.27 (±19.72)	389.07	1.0	0.90	4.04	0.16
No Pickleball + No Spencer Treatment	3.73 (±17.09)	292.21	-	-

**Table 5:** Post hoc two-sample, two-tailed t-test comparing the average change in shoulder extension over the course of the study between the individual groups * signifies a statistically significant difference (p<0.05) Group 1: Yes Pickleball + Yes Spencer Treatment; Group 2: Yes Pickleball + No Spencer Treatment; Group 3: No Pickleball + Yes Spencer Treatment; Group 4: No Pickleball + No Spencer Treatment

Between Which Groups	1 vs. 2	1 vs. 3	1 vs. 4	2 vs. 3	2 vs. 4	3 vs. 4
p-value	0.0335*	0.0125*	0.0024*	0.25	0.09	0.71
t-value	2.27	2.69	3.38	1.18	1.80	0.38

To compare the average change in shoulder abduction ROM over the course of the study, Table 6 displays the results of an unbalanced, two-factor ANOVA with replication, and Table 7 displays post-hoc two-sample, two-tailed t-tests, comparing average change in shoulder abduction between the individual groups. The results of this ANOVA demonstrate that pickleball, as an effect by itself, did not increase abduction, regardless of concurrent treatment (p=0.1276), and Spencer treatment, as an effect by itself, did increase abduction, regardless of concurrent treatment (p=0.0330). When examining the interaction of the two treatments, the presence of pickleball did affect the Spencer treatment in a non-additive manner, and vice versa (p=0.0280), in regards to abduction. Post-hoc t-tests revealed that a difference was present between the P+T group (average change of 20.67 (±16.27) degrees of abduction) versus P+NT group (average change of 2.18 (±13.09) degrees of abduction), a difference of 18.49 degrees, p=0.0071; P+T group (average change of 20.67 (±16.27) degrees of abduction) versus NP+T group (average change of 6.00 (±15.40) degrees of abduction), a difference of 14.67 degrees, p=0.0243; P+T group (average change of 20.67 (±16.27) degrees of abduction) versus NP+NT group (average change of 5.20 (±11.16) degrees of abduction), a difference of 15.47 degrees, p=0.0072. No significant difference in the average change of degrees of shoulder abduction was found between any of the other groups. These findings demonstrate a significant difference in the average change of degrees of shoulder abduction between the P+T group compared to all three other groups. This suggests that exercise involving the glenohumeral joint with recreational pickleball in combination with the use of Spencer treatment shows significantly greater improvements in degrees of shoulder abduction compared to playing recreational pickleball, or receiving Spencer treatment in isolation, or receiving neither intervention.

**Table 6:** Results of an unbalanced, two-factor ANOVA with replication analyzing each intervention’s impact and interaction on shoulder abduction * signifies a statistically significant difference (p<0.05); SD: standard deviation; ANOVA: analysis of variance

Group	Average Change of Abduction (Degrees) Over the Course of Study	Variance	df	F	F-crit	Effect P-value
Yes Pickleball + Yes Spencer Treatment	20.67 (±16.27)	264.61	1.00	2.40	4.04	0.0280*
Yes Pickleball + No Spencer Treatment	2.18 (±13.09)	171.36	1.00	4.80	4.04	0.1276
No Pickleball + Yes Spencer Treatment	6.0 (±15.40)	237.29	1.00	5.14	4.04	0.0330*
No Pickleball + No Spencer Treatment	5.2 (±11.16)	124.60		

**Table 7:** Post hoc two-sample, two-tailed t-test comparing the average change in shoulder abduction over the course of the study between the individual groups * signifies a statistically significant difference (p<0.05). Group 1: Yes Pickleball + Yes Spencer Treatment; Group 2: Yes Pickleball + No Spencer Treatment; Group 3: No Pickleball + Yes Spencer Treatment; Group 4: No Pickleball + No Spencer Treatment

Between Which Groups	1 vs. 2	1 vs. 3	1 vs. 4	2 vs. 3	2 vs. 4	3 vs. 4
p-value	0.0071*	0.0243*	0.0072*	0.51	0.53	0.87
t-value	2.98	2.40	2.93	-0.66	-0.63	0.16

To compare the average change in shoulder adduction ROM over the course of the study, Table 8 displays the results of an unbalanced, two-factor ANOVA with replication, and Table 9 displays post-hoc two-sample, two-tailed t-tests, comparing average change in shoulder adduction between the individual groups. The results of this ANOVA demonstrate that pickleball, as an effect by itself, did not increase adduction, regardless of concurrent treatment (p=0.4520), and Spencer treatment, as an effect by itself, did not increase adduction, regardless of concurrent treatment (p=0.3960). When examining the interaction of the two treatments, the presence of pickleball did not affect the Spencer treatment in a non-additive manner, and vice versa (p=0.9737), in regards to adduction. Post-hoc t-tests revealed that no significant differences in average change in degrees of shoulder adduction were found between any of the four groups. This suggests that exercise involving the glenohumeral joint with recreational pickleball in combination with the use of Spencer treatment does not show any significant difference in average change in degrees of shoulder adduction compared to playing recreational pickleball, or receiving Spencer treatment in isolation, or receiving neither intervention.

**Table 8:** Results of an unbalanced, two-factor ANOVA with replication analyzing each intervention’s impact and interaction on shoulder adduction SD: standard deviation; ANOVA: analysis of variance

Group	Average Change of Adduction (Degrees) Over the Course of Study	Variance	df	F	F-crit	Effect P-value
Yes Pickleball + Yes Spencer Treatment	7.75 (±16.03)	256.93	1.00	0.58	4.04	0.9737
Yes Pickleball + No Spencer Treatment	4.36 (±11.34)	128.65	1.00	0.74	4.04	0.4520
No Pickleball + Yes Spencer Treatment	4.73 (±17.13)	293.50	1.00	0.00	4.04	0.3960
No Pickleball + No Spencer Treatment	1.60 (±8.72)	75.97	-	-

**Table 9:** Post hoc two-sample, two-tailed t-test comparing the average change in shoulder adduction over the course of the study between the individual groups Group 1: Yes Pickleball + Yes Spencer Treatment; Group 2: Yes Pickleball + No Spencer Treatment; Group 3: No Pickleball + Yes Spencer Treatment; Group 4: No Pickleball + No Spencer Treatment

Between Which Groups	1 vs. 2	1 vs. 3	1 vs. 4	2 vs. 3	2 vs. 4	3 vs. 4
p-value	0.57	0.64	0.21	0.95	0.49	0.53
t-value	0.58	0.47	1.27	-0.06	0.70	0.63

To compare the average change in shoulder internal rotation ROM over the course of the study, Table 10 displays the results of an unbalanced, two-factor ANOVA with replication, and Table 11 displays post-hoc two-sample, two-tailed t-tests, comparing average change in shoulder internal rotation between the individual groups. The results of this ANOVA demonstrate that pickleball, as an effect by itself, did not increase internal rotation, regardless of concurrent treatment (p=0.2320), and Spencer treatment, as an effect by itself, did not increase internal rotation, regardless of concurrent treatment (p=0.1400). When examining the interaction of the two treatments, the presence of pickleball did not affect the Spencer treatment in a non-additive manner, and vice versa (p=0.1100), in regards to internal rotation. Post-hoc t-tests revealed that a difference was present between the P+T group (average change of 27.08 (±18.57) degrees of internal rotation) versus P+NT group (average change of 4.27 (±19.35) degrees of internal rotation), a difference of 22.81 degrees, p=0.0089; P+T group (average change of 27.08 (±18.57) degrees of internal rotation) versus NP+NT group (average change of 7.40 (±24.92) degrees of internal rotation), a difference of 19.68 degrees, p=0.0318. No significant differences in average change in degrees of shoulder internal rotation were found between any of the other groups. These findings demonstrate a significant difference in degrees of shoulder internal rotation between the P+T group compared to the P+NT group and the NP+NT group. This suggests that exercise involving the glenohumeral joint with recreational pickleball in combination with the use of Spencer treatment shows significantly greater improvements in degrees of shoulder internal rotation compared to playing recreational pickleball in isolation, or receiving neither intervention.

**Table 10:** Results of an unbalanced, two-factor ANOVA with replication analyzing each intervention’s impact and interaction on shoulder internal rotation SD: standard deviation; ANOVA: analysis of variance

Group	Average Change of Internal Rotation (Degrees) Over the Course of Study	Variance	df	F	F-crit	Effect P-value
Yes Pickleball + Yes Spencer Treatment	27.08 (±18.57)	344.99	1.00	1.46	4.04	0.1100
Yes Pickleball + No Spencer Treatment	4.27 (±19.35)	374.42	1.00	2.25	4.04	0.2320
No Pickleball + Yes Spencer Treatment	7.93 (±31.26)	977.21	1.00	2.64	4.04	0.1400
No Pickleball + No Spencer Treatment	7.40 (±24.92)	621.11	-	-

**Table 11:** Post hoc two-sample, two-tailed t-test comparing the average change in shoulder internal rotation over the course of the study between the individual groups * signifies a statistically significant difference (p < 0.05). Group 1: Yes Pickleball + Yes Spencer Treatment; Group 2: Yes Pickleball + No Spencer Treatment; Group 3: No Pickleball + Yes Spencer Treatment; Group 4: No Pickleball + No Spencer Treatment

Between Which Groups	1 vs. 2	1 vs. 3	1 vs. 4	2 vs. 3	2 vs. 4	3 vs. 4
p-value	0.0089*	0.07	0.0318*	0.74	0.73	0.96
t-value	2.88	1.87	2.27	-0.34	-0.35	0.05

To compare the average change in shoulder external rotation ROM over the course of the study, Table 12 displays the results of an unbalanced, two-factor ANOVA with replication, and Table 13 displays post-hoc two-sample, two-tailed t-tests, comparing comparing average change in shoulder external rotation. The results of this ANOVA demonstrate that pickleball, as an effect by itself, did not increase external rotation, regardless of concurrent treatment (p=0.0525), and Spencer treatment, as an effect by itself, did not increase external rotation, regardless of concurrent treatment (p=0.1490). When examining the interaction of the two treatments, the presence of pickleball did not affect the Spencer treatment in a non-additive manner, and vice versa (p=0.8190) in regards to external rotation. Post-hoc t-tests revealed that a difference was present between the P+T group (average change of 17.83 (±10.26) degrees of external rotation) versus the NP+NT group (average change of 4.20 (±8.90) degrees of external rotation), a difference of 13.63 degrees, p=0.0011. No significant differences in average change in degrees of shoulder external rotation were found between any of the other groups. These findings demonstrate a significant difference in average change in degrees of shoulder external rotation between the P+T group compared to the NP+NT group. This suggests that exercise involving the glenohumeral joint with recreational pickleball, with concurrent use of Spencer treatment, shows significantly greater improvements in degrees of shoulder external rotation compared to abstaining from both pickleball and Spencer treatment simultaneously.

**Table 12:** Results of an unbalanced, two-factor ANOVA with replication analyzing each intervention’s impact and interaction on shoulder external rotation SD: standard deviation; ANOVA: analysis of variance

Group	Average Change of External Rotation (Degrees) Over the Course of Study	Variance	df	F	F-crit	Effect P-value
Yes Pickleball + Yes Spencer Treatment	17.83 (±10.26)	105.24	1.00	3.95	4.04	0.8190
Yes Pickleball + No Spencer Treatment	11.09 (±15.00)	225.09	1.00	2.15	4.04	0.0525
No Pickleball + Yes Spencer Treatment	9.13 (±19.55)	382.12	1.00	0.05	4.04	0.1490
No Pickleball + No Spencer Treatment	4.20 (±8.90)	79.17	-	-

**Table 13:** Post hoc two-sample, two-tailed t-test comparing the average change in shoulder external rotation over the course of the study between the individual groups * signifies a statistically significant difference (p < 0.05). Group 1: Yes Pickleball + Yes Spencer Treatment; Group 2: Yes Pickleball + No Spencer Treatment; Group 3: No Pickleball + Yes Spencer Treatment; Group 4: No Pickleball + No Spencer Treatment

Between Which Groups	1 vs. 2	1 vs. 3	1 vs. 4	2 vs. 3	2 vs. 4	3 vs. 4
p-value	0.22	0.18	0.0011*	0.78	0.16	0.38
t-value	1.27	1.39	3.70	0.28	1.47	0.89

Summary of significant findings

Statistically significant differences were found amongst the four different groups in terms of average change in degrees of shoulder flexion, extension, abduction, internal rotation, and external rotation over the two-week course of the study. This difference was seen only when comparing the P+T group, which experienced greater change, to each of the other three groups in flexion, extension, and abduction. In regard to internal rotation, this difference was seen only when comparing the P+T group, which experienced greater change, to the P+NT and NP+NT (control) groups. In regard to external rotation, this difference was only seen when comparing the P+T group, which experienced greater change, to the NP+NT (control) group. No statistically significant differences were observed amongst the four different groups in regard to the average change in degrees of shoulder adduction over the two-week course of the study. When examining the interaction between the two interventions during their concurrent administration, the two interventions affected each other in a non-additive manner only when examining abduction. For all other motions, the two interventions did not affect each other in a non-additive manner.

## Discussion

This study demonstrates that playing recreational pickleball in combination with receiving the Spencer technique of the shoulder (P+T group) results in significantly greater improvements in shoulder flexion, extension, and abduction when compared to either intervention alone or no intervention at all. When examining internal rotation, this difference was seen when comparing the P+T group, which experienced greater change, to the P+NT and NP+NT groups. For external rotation, this difference was seen when comparing the P+T group, which experienced greater change, to the NP+NT group. These findings highlight the benefit of combining recreational exercise involving active shoulder movement, such as recreational pickleball, and manual osteopathic therapy in promoting glenohumeral joint mobility.

The significantly greater improvement seen in the P+T group, when compared to the other groups, aligns with prior evidence demonstrating that combining manual therapy with therapeutic exercise creates superior outcomes in shoulder rehabilitation [14-18]. A myriad of studies have demonstrated that manual therapy alone may address capsular tightness, soft tissue restrictions, and joint adhesions, while active joint movement through exercise reinforces these functional gains and augments the benefits of treatment [14-18]. In a study by Tauqeer et al., it was found that manual therapy, in addition to stretching and strengthening exercises, yielded superior benefits in pain reduction, scapular ROM, and functional capacity compared to exercise therapy alone in patients with shoulder impingement syndrome [14]. A systematic review by Yang et al. further concluded that manual therapy combined with exercise therapy significantly reduced pain and improved physical function compared to exercise alone, particularly in populations with shoulder dysfunctions such as adhesive capsulitis and rotator cuff tendinopathy [15]. Manual joint mobilization likely temporarily improves capsular compliance, and it can be speculated that following treatment with recreational exercise involving joint movement may help reinforce these gains by further engaging the joint structures and promoting longer-lasting improvements [19]. This combined treatment approach has shown success across various rehabilitation settings, including the treatment of shoulder impingement syndrome, adhesive capsulitis, and, in this study, healthy individuals with no underlying shoulder pathologies [14-18].

As a specific form of manual therapy, the Spencer technique has been shown to effectively improve capsular mobility and shoulder function [3,4,20]. Previous research has shown its benefits in active populations, including a controlled trial by Curcio et al. that demonstrated significant improvements in shoulder abduction and internal rotation in collegiate baseball players following Spencer treatment [2]. However, while the Spencer technique alone can be successful at addressing mechanical and myofascial restrictions, our study shows that its impact may be limited without supplementing treatment with active joint movement via recreational exercise. Participants who received the Spencer technique in isolation (NP+T group) did not see a significant difference in ROM change from baseline compared to the double-negative control group (NP+NT) in any of the six planes of motion tested. These results strengthen the argument that using the Spencer technique in combination with recreational exercise involving active shoulder movement can optimize ROM improvements, especially in flexion, extension, and abduction. The repeated functional demands of pickleball likely served to reinforce joint mobilization gains, leading to significant ROM improvements, as described in prior studies evaluating combined functional movement and manual therapy approaches [19].

The absence of significant differences between the four groups in adduction may be due to several factors. The biomechanical demands of pickleball primarily emphasize repetitive overhead swinging motions, which may disproportionately affect certain planes of motion [21]. To see significant differences between the four groups in adduction longer intervention periods may be required to yield measurable differences, particularly in the relatively small sample size used in this study.

Several limitations of this study should be noted. First, our cohort consisted of healthy, asymptomatic volunteers, which may limit generalizability to patients with active shoulder pathology and pathologic ROM restrictions. The two-week intervention period may also underestimate the full potential of the ROM gains, possibly seen with a longer treatment course. Additionally, while compliance and participation rates were high, pickleball play in terms of skill, intensity, and duration of each session was not standardized and likely introduced variability in playing sessions. The relatively small sample size (n=53) also limits statistical power, and all ROM measurements were obtained using hand-held goniometers, which are inherently subject to some variability despite attempts to standardize measurement technique.

Future studies should explore the efficacy of this combined approach in clinical populations, particularly those with shoulder stiffness or pathologic ROM restrictions. Long-term retention of ROM gains should also be further investigated. Future investigators should incorporate larger sample sizes and utilize more precise measurement tools, such as motion capture systems, to enhance measurement accuracy and reliability. Additionally, comparative studies investigating alternative exercise and active joint mobility interventions, such as swimming, resistance training, or other racquet sports, such as tennis, may further clarify the unique contribution of dynamic activities like pickleball in improving glenohumeral joint mobility.

## Conclusions

This study demonstrates that the combination of recreational pickleball participation and Spencer technique treatment produces significantly greater improvements in shoulder flexion, extension, and abduction, compared to either intervention in isolation or no intervention at all. Significant ROM improvements were seen in the P+T group compared to other specific intervention groups in internal rotation and external rotation as well. These findings highlight the potential benefit of combining targeted manual osteopathic therapy, such as the Spencer technique, with dynamic, functional movement activities, such as pickleball, in optimizing shoulder mobility. This integrative approach offers a promising, non-invasive strategy for both preventive care and rehabilitation of shoulder dysfunction. Additional studies are warranted to further explore the interconnected nature of OMT and recreational exercise in improving joint function. Future studies should include larger sample sizes, extend intervention periods, incorporate a variety of recreational activities, and examine the effects in patients with diverse shoulder pathologies. With a more complete understanding of how OMT affects different patient populations with varying exercise habits, osteopathic clinicians will be better positioned to deliver more personalized care to ensure optimal clinical outcomes.
